# Transforming Cultural Ideas of Aging and Disability to Improve Policy and Practice

**DOI:** 10.1093/geroni/igaa057.2217

**Published:** 2020-12-16

**Authors:** Anne Ordway

**Affiliations:** U.S. Department of Health and Human Services, Washington, District of Columbia, United States

## Abstract

Aging and disability are normative processes that extend across the lifespan. However, ageism and ableism are incorporated into many of our practices, programs, and policies—devaluing the lives of older adults and people aging with disabilities and ultimately preventing their full participation in society. Ageism and ableism are closely connected. For example, both systems identify impairment as an individual and social liability. As recent studies have demonstrated, this has real world implications for the quantity and quality of health care requested, delivered, and received by both older adults and people with disabilities. In this session, we discuss the connections between these two forms of oppression and present recent work by researchers in both fields and the FrameWorks Institute that shows how to transform our cultural ideas of aging and disability and development more inclusive policies and services. Part of a symposium sponsored by the Lifelong Disabilities Interest Group.

